# An Open Letter to Dr. Hill from Dr. Daniel Parker

**Published:** 1899-11

**Authors:** Daniel Parker

**Affiliations:** Calvert, Texas


### Correspondence.

### An Open Letter to Dr. Hill from Dr. Daniel Parker.

CALVERT, TEXAS, October 19, 1899.

*Dr. J. S. Hill, Greenville, Texas.*

DEAR SIR: I have just received yours of the 12th inst., in which you state that "a branch hospital of the Narcotic Hospital Company of St. Louis, has been established at Greenville, Texas, with myself (yourself) in charge." This is followed by the statement that "we have an infallible cure for the morphine, opium, cocaine, whiskey or tobacco habits." Then comes an "absolute guaranty" of "permanent relief," reference to endorsements, an appeal for the names of such unfortunate victims of the habits mentioned as I may know, etc. A card accompanies this letter, which contains the remarkable statement that you *"can cure anything that walks the earth* of narcotic habits."

In reply to this I will say, that I shall send you no names, for at least two reasons.

In the first place, I do not believe that you have any "infallible cure" for the habits mentioned; and, in the second place, I should be sorry to know that any already unfortunate person had fallen into your hands through any act of mine.

The passion for stimulants and narcotics has filled our prisons with felons, the hearts of multiplied thousands of women and children with sorrow, shame and despair, and has so rent the souls of the victims themselves with the tortures of thirst and the pangs of disgrace, that they often seek relief in death.

If you really believe that you have a remedy for any of these thrice cursed habits, and are withholding a knowledge of it for the sake of profit to yourself, God help those who fall into your hands.

If you do not believe what you say when you claim an "infallible cure," quit writing to reputable physicians, put your picture and advertisements in the papers and go into quackery right, like the "Spermatorrhœa," "Celery Compound," "Fistula" and "Peruna" folks. Work in your own class. The physicians of Texas are not going to send any grist to your mill.

Yours,

DANIEL PARKER.

